# Transcriptome Profile of the Green Odorous Frog (*Odorrana margaretae*)

**DOI:** 10.1371/journal.pone.0075211

**Published:** 2013-09-20

**Authors:** Liang Qiao, Weizhao Yang, Jinzhong Fu, Zhaobin Song

**Affiliations:** 1 Sichuan Key Laboratory of Conservation Biology on Endangered Wildlife, College of Life Sciences, Sichuan University, Chengdu, China; 2 Chengdu Institute of Biology, Chinese Academy of Sciences, Chengdu, China; 3 Department of Integrative Biology, University of Guelph, Guelph, Ontario, Canada; 4 Key Laboratory of Bio-Resources and Eco-Environment of Ministry of Education, College of Life Sciences, Sichuan University, Chengdu, China; University of Lausanne, Switzerland

## Abstract

Transcriptome profiles provide a practical and inexpensive alternative to explore genomic data in non-model organisms, particularly in amphibians where the genomes are very large and complex. The odorous frog *Odorrana* *margaretae* (Anura: Ranidae) is a dominant species in the mountain stream ecosystem of western China. Limited knowledge of its genetic background has hindered research on this species, despite its importance in the ecosystem and as biological resources. Here we report the transcriptome of *O* *. margaretae* in order to establish the foundation for genetic research. Using an Illumina sequencing platform, 62,321,166 raw reads were acquired. After a *de novo* assembly, 37,906 transcripts were obtained, and 18,933 transcripts were annotated to 14,628 genes. We functionally classified these transcripts by Gene Ontology (GO) and Kyoto Encyclopedia of Genes and Genomes (KEGG). A total of 11,457 unique transcripts were assigned to 52 GO terms, and 1,438 transcripts were assigned to 128 KEGG pathways. Furthermore, we identified 27 potential antimicrobial peptides (AMPs), 50,351 single nucleotide polymorphism (SNP) sites, and 2,574 microsatellite DNA loci. The transcriptome profile of this species will shed more light on its genetic background and provide useful tools for future studies of this species, as well as other species in the genus *Odorrana*. It will also contribute to the accumulation of amphibian genomic data.

## Introduction

Genomics has revolutionized many disciplines of biological science, and genomic data of non-model organisms are rapidly accumulating [1-3]. Among vertebrates, for example, a total of 61 annotated genomes are currently available from Ensembl database (June 21, 2013). Nevertheless, amphibians, as a major transitional group in the evolutionary history of vertebrates, are currently represented by only one species ( *Xenopus* *tropicalis* ) [4]. This is likely due to the fact that amphibians generally have very large and complex genomes [5,6]. As a small but important part of the genome, transcriptome includes most protein coding genes. With advances of the Next Generation Sequencing (NGS) technologies, transcriptome data offer an opportunity to deliver fast, inexpensive, and accurate genome information for genomic exploration in non-model organisms [7,8]. Transcriptome data are particularly useful for amphibians, because of their large genome sizes, which make obtaining whole genome data difficult. Currently, nine amphibian transcriptomes have been reported, including *Xenopus* *tropicalis* [9], *Cycloranaalboguttata* [10], *Rana* *chensinensis* [11,12], *R* *. kukunoris* [11], *R* *. muscosa* [13], *R* *. sierra* [13], *Hyla arborea* [14], *Notophthalmus* *viridescens* [15,16], and *Ambystoma* *mexicanum* [17]. The accumulation of transcriptome data will provide a ‘sneak peek’ of amphibian genome evolution.

The green odorous frog *Odorrana* *margaretae* is a typical anuran from the family Ranidae, and is an important species both ecologically and in terms of biological resources. It is a dominant species in the stream ecosystem of the Hengduan Mountain, a global diversity hotspot [18], and is an excellent environmental indicator species. Odorous frog species are sensitive to environmental changes, and at least two of them have been observed to shift their ranges northward possibly as a response to global warming ([19], personal observation). Additionally, odorous frogs are reservoirs for antimicrobial peptides (AMPs), and may represent the most extreme AMPs diversity in nature [20]. AMPs are generally short peptides with potent antibacterial and antifungal activity [21]. So far, 728 different AMPs have been identified from nine odorous frog species, which account for approximately 30% of all AMPs discovered [20].

A transcriptome profile of the green odorous frog will provide new molecular markers for ecological research of this as well as other odorous frog species. Single nucleotide polymorphism (SNP) and microsatellite DNA loci are excellent genetic markers, and are commonly employed in population genetic and molecular ecological studies. They are abundant in transcriptomes. Furthermore, due to limitations of conventional biochemical isolation methods, some peptides cannot be isolated and purified. Transcriptome data will not suffer from these limitations and provide an important alternative way to identify potential AMPs.

In this study, we construct the transcriptome profile of *O* *. margaretae* using an Illumina sequencing platform. Multiple tissues types from multiple individuals were pooled to maximize the chance of revealing as many genes as possible. After *de novo* assembly, we implemented a functional annotation using bioinformatic analysis. In addition, we made a genome wide search for cDNA encoding AMPs, microsatellite DNA loci, and SNP sites from the transcriptome. Our data will serve as an important step forward to establish the foundation for genomic research of amphibians, as well as to provide new markers for molecular ecological studies.

## Results

### Illumina sequencing, *de novo* assembly, and gene annotation

**Figure 1:**
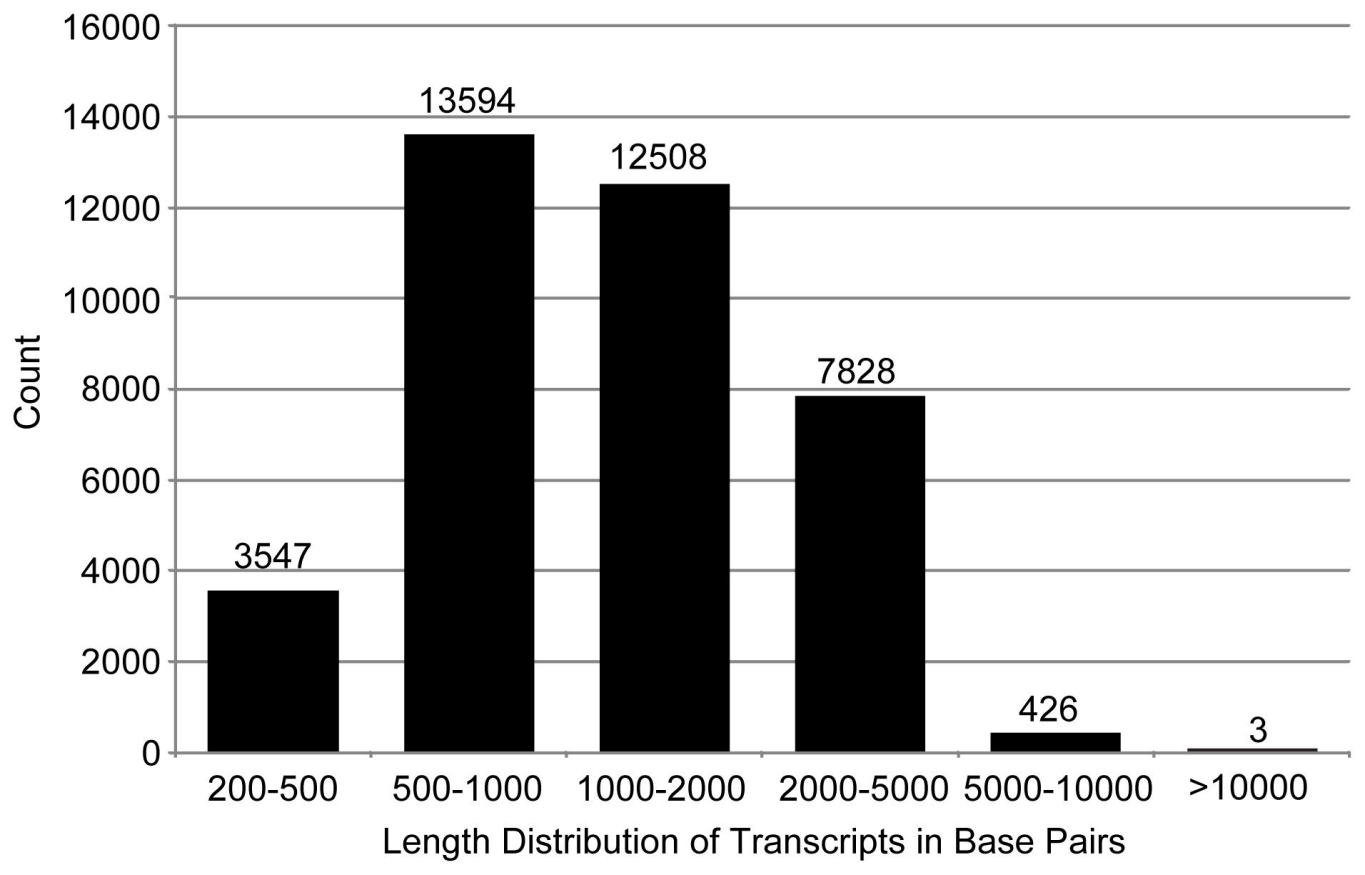
Length distribution of transcripts in base pairs. The numbers of transcripts are shown on top of each bar.

Illumina sequencing of *O* *. margaretae* yielded a total of 62,321,166 raw reads. Among them, 3,504,433 were first filtered out before assembly as low quality sequences or potential contaminations. Thirty combinations of multiple K-mer lengths and coverage cut-off values were used to perform *de novo* assembly of the clean reads [22,23]. Finally, we merged the 30 raw assemblies by integrating sequence overlaps and eliminating redundancies. The final assembly included a total of 54.3 mega base pairs (Mb), and 37,906 transcripts were obtained with a N50 length of 1,870 base pairs (bps) and a mean length of 1,434 bps. The sequencing information is presented in Table 1 and the length distribution of all transcripts is shown in Figure 1. All sequence reads are deposited at NCBI (accession number SRA091981), and the final assembly is presented as supporting information (Sequence S1 and S2).

**Table 1:** Summary of transcriptome data for *Odorrana* *margaretae* .

Total number of raw reads	62,321,166
Total number of clean reads	58,816,733
Length of reads (bp)	101
Total length of clean reads	5.88G
Total length of assembly (bp)	54,362,822
Total number of transcripts	37,906
N50 length of assembly (bp)	1,870
Mean length of assembly (bp)	1,434
Median length of assembly (bp)	1,096
Transcripts annotated	18,933
Number of unique genes represented	14,628

bp = base pair

**Figure 2:**
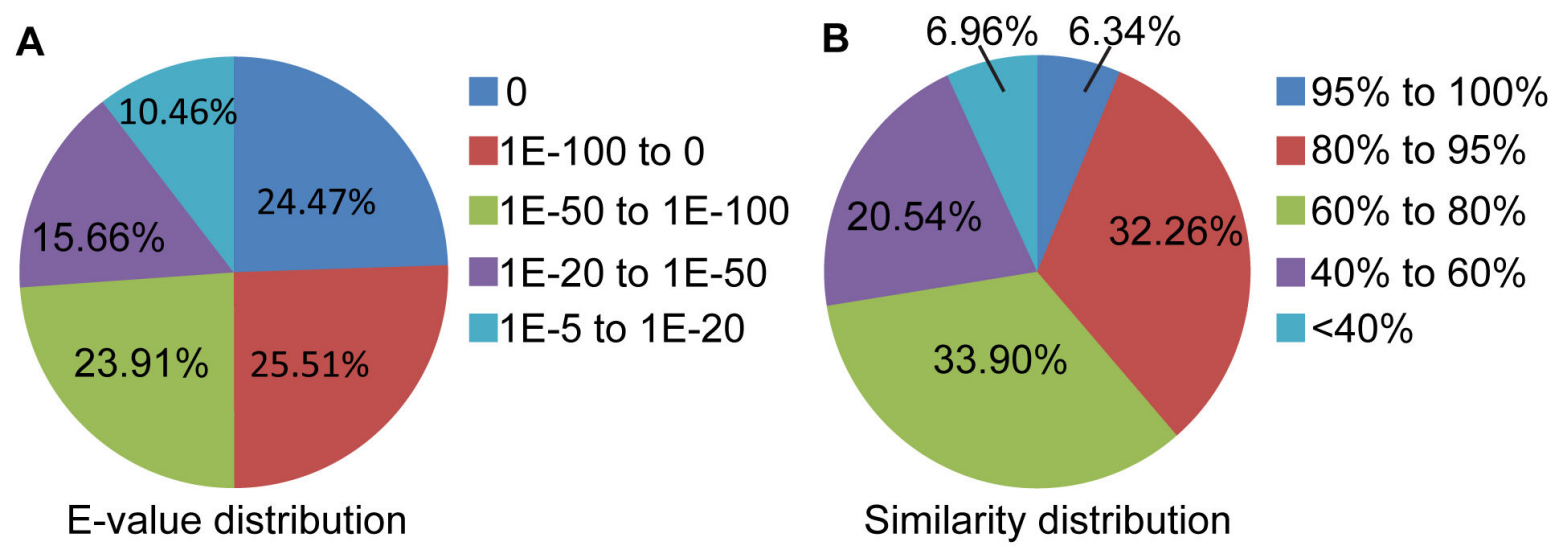
Characteristics of gene annotation of assembled transcripts against the reference dataset. (A) E-value distribution of BLASTX hits for transcript with a cut-off E-value of 1E-5. (B) Similarity distribution of BLASTX hits for transcript.

A reference dataset was constructed for gene annotation, which included protein data from seven species ( *Anolis* *carolinensis* , *Danio rerio*, *Gallus gallus*, *Homo sapiens*, *Mus musculus*, *Oryzias latipes*, and *Xenopus* *tropicalis* ), representing all major lineages of vertebrates. Transcripts were blasted against this dataset. In total, 18,933 transcripts were annotated to 14,628 genes, which comprised 49.95% of the total transcripts. E-value distribution showed that 73.89% of the annotated sequences had strong homology (E-value below 1E-50), and similarity distribution showed that 72.50% of the annotated sequences had a similarity greater than 60% (Table 1 and Figure 2).

### GO classification

**Figure 3:**
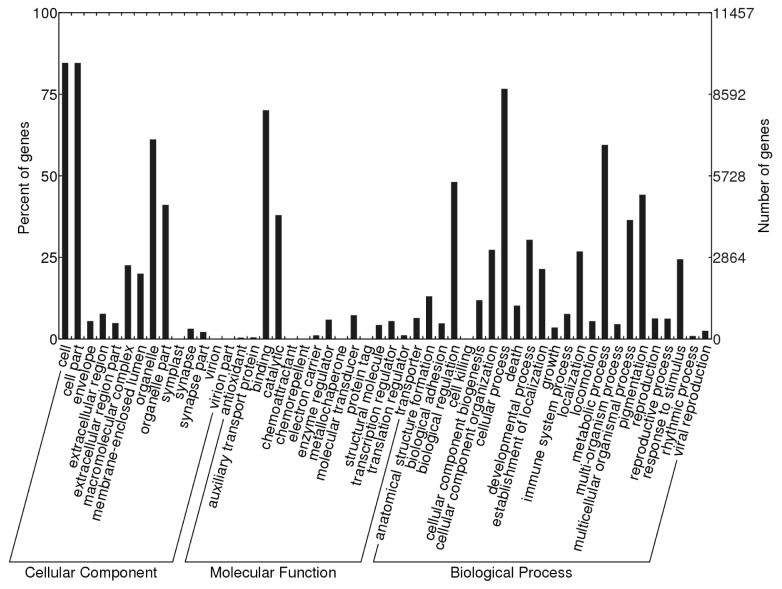
Distribution of Gene Ontology (GO) categories (level 2) of transcripts for *O* *. margaretae* . GO functional annotations are summarized in three main categories: cellular component, molecular function and biological process.

Gene Ontology (GO) is widely used to standardize representation of genes across species and provides a set of structured and controlled vocabularies for annotating genes, gene products, and sequences [24]. In total, 11,457 unique transcripts were assigned to 52 level-2 GO terms, which were summarized under three main GO categories, including cellular component, molecular function, and biological process (Figure 3). Compared to the GO annotations of two other species of the same family, *R* *. chensinensis* and *R* *. kukunoris* , the GO category distributions of the transcripts for the three ranid frogs were highly similar (Figure S1). Within the GO category of cellular components, 14 level-2 categories were identified, and the terms cell, cell part, and organelle were the most abundant (>50%). Within the GO category of molecular function, 15 level-2 categories were identified, and the term binding was the most abundant (>50%). For biological process function, 23 level-2 categories were identified, and the terms of cellular process and metabolic process were the most abundant (>50%).

### KEGG analysis

**Figure 4:**
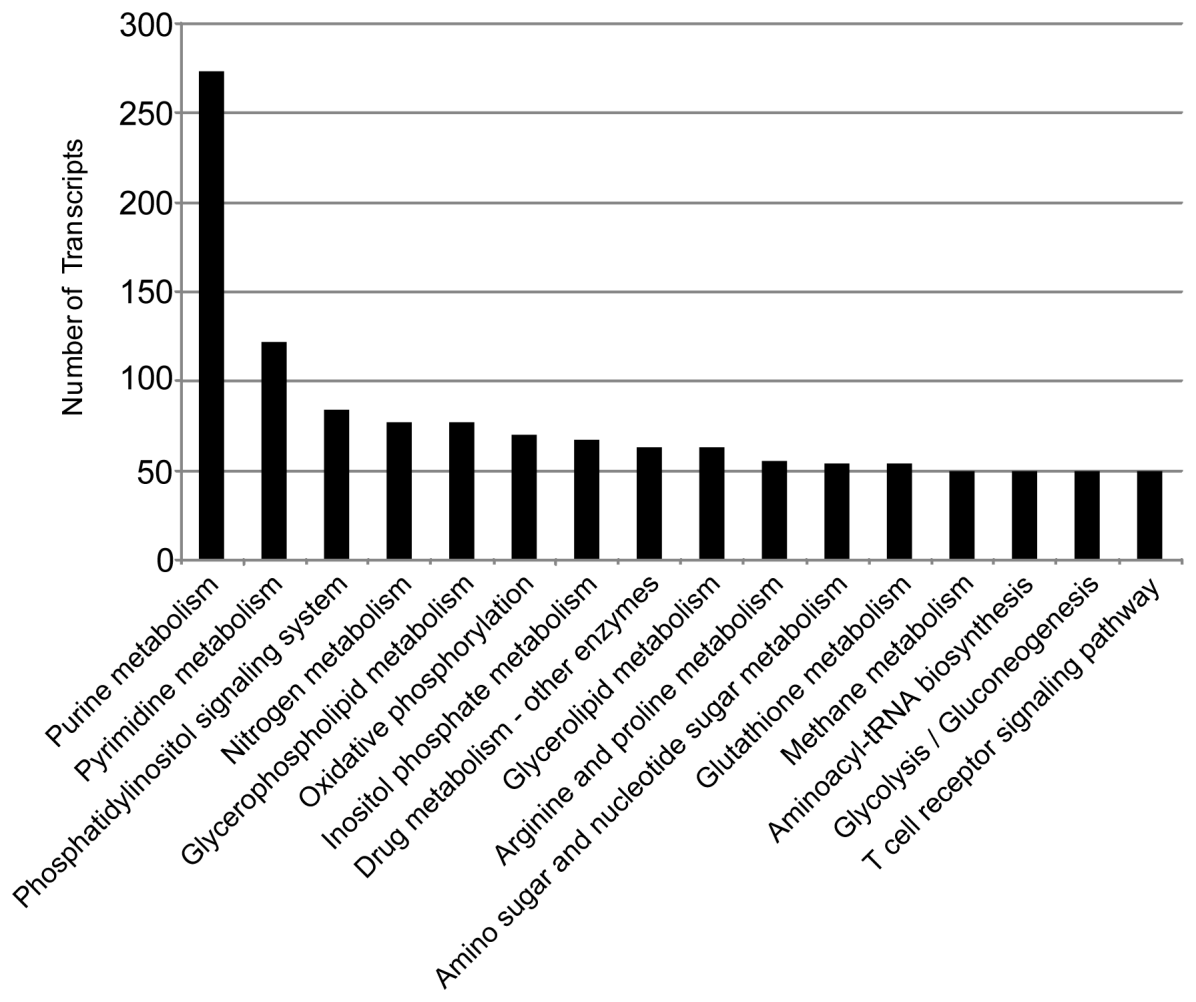
Distribution of *O* *. margaretae* transcripts among Kyoto Encyclopedia of Genes and Genomes (KEGG) pathways. The top 16 most highly represented pathways are shown. Analysis was performed using Blast2GO and the KEGG database.

Kyoto Encyclopedia of Genes and Genomes (KEGG) [25] database was used to identify potential biological pathways represented in the *O* *. margaretae* transcriptome. A total of 1,438 transcripts were assigned to 128 KEGG pathways (Figure 4, Table S1). Among the pathways, purine metabolism, pyrimidine metabolism, phosphatidylinositol signaling system, and a few others were highly represented. These annotations provide a valuable resource for investigating specific processes, functions, and pathways in amphibian research.

### Comparison to other amphibian transcriptomes

Overall, our data were similar to other reported amphibian transcriptomes from Illumina sequencing platform (Table 2). We had a slightly longer N50 length than most of the other transcriptomes, suggesting the quality of our assembly was high. Clearly, our strategy with multiple K-mer length and cut-off value combinations worked well. The transcriptomes of *R* *. chensinensis* [12] and *Hyla arborea* [14] had short N50 lengths and large numbers of transcripts, suggesting that the assembly generated many short transcripts and could be improved. Among the eight transcriptomes, *C* *. alboguttata* had the largest number of transcripts; this is not particularly surprising, considering that it also had the largest number of raw reads. In terms of transcript annotation rate, *O* *. margaretae* had approximately 50% transcripts that were annotated, whereas the rates were approximately 33%, 40%, 44%, 42% and 32% for *C* *. alboguttata* [10], *R* *. chensinensis* [11], *R* *. chensinensis* [12], *R* *. kukunoris* [11], and *N* *. viridescens* [15], respectively.

**Table 2:** Comparison of transcriptome data for six amphibian species.

	Number of raw reads	Number of transcripts	N50 length of assembly (bp)	Transcripts annotated
* Odorrana margaretae *	62,321,166	37,906	1,870	18,933
*Cycloranaalboguttata* [10]	400,032,568	68,947	1,586	22,695
*Rana* *chensinensis* [11]	67,676,712	41,858	1,333	16,738
*Rana* *chensinensis* [12]	39,300,002	78,117	413	34,706
*Rana* *kukunoris* [11]	66,476,534	39,293	1,485	16,549
*Hyla arborea* [14]	11,034,721	83,293	700	-
*Notophthalmus* *viridescens* [15]	-	120,922	975	38,384
*Notophthalmus* *viridescens* [16]	-	118,893	2,016	-

bp=base pair; - =data not available

### AMPs

A total of 27 transcripts were identified as potential AMPs (Table S2). Among them, five transcripts matched AMPs that were previously identified in *O* *. margaratae* , including Esculentin-2-OMar1, Brevinin-2-OMar1, Odorranain-A-Omar1, Esculentin-l-OMar4, and Margaratain-C1. The other 22 were new to *O* *. margaratae* . Although the exact functions of these peptides remain largely unknown, AMPs have received much attention lately [20,26], due to their anti-infective properties.

### Molecular markers

A total of 50,351 single nucleotide polymorphism (SNP) sites were identified; 33,735 were transitions and 16,616 were transversions (Table S3). We also identified 2,574 microsatellite DNA loci, of which 78.59% were dinucleotide repeats, 19.54% were trinucleotide repeats, 1.40% were tetranucleotide repeats, 0.43% were pentanucleotide repeats, and 0.04% were hexanucleotide repeats (Table S4). These molecular markers will provide useful tools in population genetic and molecular ecological studies of *O* *. margaretae* , and potentially for other odorous frogs as well.

## Discussion

Available genomic data for amphibians are very limited and our transcriptome data of *O* *. margaretae* will certainly make a significant contribution to the understanding of genome evolution of amphibians. Currently, there is only one completed genome sequence for amphibians (i.e. *X* *. tropicalis* ) available in public databases [4]. Amphibians often have large genome sizes (0.95-120.60 pg [6]), which makes genome sequencing and analysis difficult. With the current technological limitations, transcriptome data provide a viable alternative to whole genome sequencing. Although only representing a small portion of the genome, transcriptome includes most protein coding genes and arguably represent the most functional part of the genome. The accumulation of transcriptome data will not only offer us an opportunity to have a glance at the genome evolution of amphibians, but also establish foundation for gene expression level genomic studies. There are currently nine published amphibian transcriptomes [9-17]. More data are needed to establish patterns and formulate meaningful hypotheses.

We obtained a high quality *de novo* assembly by using a multiple K-mer lengths and cut-off values strategy. The N50 length of our assembly is higher than most other amphibian transcriptome data without a significant reduction of the overall number of unique transcripts (Table 2). N50 length is commonly used for assembly evaluation, and a higher number suggests high quality assembly [27]. In addition, our high transcripts annotation rate also indicates that the accuracy of assembled transcripts is greater than those assembled in other amphibians (Table 2). Clearly, our strategy worked well in this case.

We only identified a small number of AMPs. Previously, 72 mature AMPs have been identified in *O* *. margaratae* by peptidomic analysis [20], and we only found five homologous to these 72. This is most likely due to the fact that we did not include skin tissue samples in our RNA extraction, which is often a rich resource for AMPs [21]. Nevertheless, our study demonstrated that AMPs not only exist in the amphibian skin, but also in other tissues, such as stomach and brain, which is consistent with several previous studies [28-30]. In addition, we identified 22 new AMPs for *O* *. margaratae* , and some of them have been identified in the skin of other *Odorrana* species, such as Brevinin-2-Omar1 and Odorranain-A-Omar1 [31]. This suggests that transcriptome is a valid alternative way for AMP discovery.

We identified a large number of microsatellite DNA and SNP loci for *O* *. margaratae* . In comparison to conventional methods for microsatellite DNA isolation (e.g. FIASCO protocol [32]), transcriptomes and NGS provide a fast, economical, and high-throughput alternative. It also selects multiple types of repeats simultaneously and is not limited by types of probes. Nevertheless, an initial characterization of these loci suggests that microsatellite DNA loci from the UTR regions generally have lower allelic diversity compared to conventionally selected loci (unpublished data). Molecular markers play an important role in contemporary biological research [33]. These microsatellite DNA loci and SNP sites will facilitate research of *O* *. margaratae* , as well as other amphibian species.

## Conclusions

Using next generation sequencing technology, we produced a transcriptome profile for an odorous frog species ( *O* *. margaretae* ). The profile is similar to other published amphibian transcriptomes. In addition, we identified 27 potential AMPs, which confirmed that NGS can serve as an alternative way for AMP discovery. A large number of microsatellite DNA loci and SNP sites were also identified, which will facilitate studies on population genetics of odorous frogs. Perhaps most importantly, our data represent a significant contribution to the accumulation of genomic data of amphibians.

## Materials and Methods

### Sample collection

In order to recover as many expressed genes as possible, multiple tissues from three adult individuals (one male and two females) and one tadpole were used. For adults, seven types of tissues (cerebrum, eye, skeletal muscle, heart, liver, testicle, and *Ootheca* ) were collected; for tadpole, the whole body was used after removing the guts. Samples of one male, one female, and the tadpole were collected from Mt. Emei (Sichuan, China; E103°38918’, N29° 56418’, 749m) in August 2012. The other female was collected from Xiaohegou Nature Reserve (Sichuan, China; E104°47257’, N32° 53149’, 1393m; approximately 345 km from the first site) in September 2012. All individuals were euthanized by immersion in MS-222 buffered solution (3g/L), and tissues were collected and stored in Sample Protector Solution (TAKARA) immediately after euthanasia. All individuals were identified by both morphological and molecular (mitochondrial cytochrome *b* gene sequence) traits.

### cDNA library construction and Illumina sequencing

Total RNA was extracted from each tissue sample individually using TRIzol® reagent (Life Technologies) and mixed with approximately same quantity. RNA integrity was assessed using the RNA 6000 Nano Assay Kit with a Bioanalyzer 2100 (Agilent Technologies) after checking the RNA purity and concentration. A single cDNA library was constructed. The mRNA was purified from total RNA using poly-T oligo-attached magnetic beads (Life Technologies). The first cDNA strand was synthesized using random oligonucleotides and M-MuLV Reverse Transcriptase (RNase H-). The second cDNA strand synthesis was subsequently performed using DNA Polymerase I and RNase H. The cDNA with an insert size of 200 bp were preferentially purified with AMPure XP beads system (Beckman Coulter) and sequenced on an Illumina HiSeq 2000 platform. 101 bp paired-end reads were generated and all raw sequence read data were stored in FastQ format. Both cDNA library construction and Illumina sequencing were performed by NovoGene (Beijing).

### Data filtration and *de novo* assembly

We first filtered the raw reads by removing the adapter sequences, reads with unknown bases call (N) more than 5%, and low quality sequences (<Q20) using an in-house workflow of Novogene and Trimmomatic [34]. Then we removed reads that were likely derived from contaminants of human and *Escherichia coli* genomes using Bow tie [35]. *De novo* assembly of clean reads was carried out using a strategy of multiple K-mer lengths and coverage cut-off values. Five different K-mer lengths (21, 31, 41, 51, and 61) and six coverage cut-off values (2, 3, 6, 10, 15, and 20) were used to generate 30 raw assemblies by ABYSS [36]. The raw assemblies were merged to produce a combined assembly. Then CD-HIT-EST [37] was used to eliminate redundancies with sequence identity threshold of 1.0 and word length of 8. CAP3 [38] was used to integrate sequence overlaps with default parameters for three times. A final assembly was generated after removing contigs shorter than 200 bp. All raw sequence reads were mapped back to the final assembly to identify variable sites using Bow tie [35] and SAMtools pipeline [39]. The base call that was consistent with the most mapped reads at a variable site was chosen for the consensus sequences using an in-house Python script.

### Gene annotation and GO/KEGG classification

We first constructed a reference dataset for gene annotation, because of the lack of genomic information of any *Odorrana* species. The dataset combined protein data of seven vertebrate species from the ENSEMBL Database [40], including *Anolis* *carolinensis* , *Danio rerio*, *Gallus gallus*, *Homo sapiens*, *Mus musculus*, *Oryzias latipes*, and *Xenopus* *tropicalis* . Assembled sequences were annotated to the reference dataset based on BLAST similarity using BLASTX [41] with an E-value cut-off of 1E-05.

GO categories and KEGG pathways were used to classify the functions and metabolic pathways of the transcripts. In order to exclude the interference from alternative splicing of transcripts, we first clustered all transcripts that matched the same reference gene; then we removed redundant transcripts and only preserved the longest transcript from each cluster to represent a unique gene. GO and KEGG classification was performed using the Blast2GO [42] pipelines with the default parameters.

### AMPs and molecular markers

In order to identify AMPs in the transcriptome of green odorous frog, we blasted the assembled transcripts against the known AMPs from Database of Anuran Defense Peptides (DADP) [43] using Blast-2.2.26+ [41] with the similarity cutoff of 80%. SNP sites were identified using SAMtools [39] pipeline after mapping all clean reads to the assembled transcripts using Bow tie with default parameters [35]. Microsatellite DNA loci were identified by QDD2 pipeline [44], and the same pipeline also automatically designed all associated primers.

### Ethics Statement

The animal specimens were collected legally. All animal collection and utility protocols were approved by the Chengdu Institute of Biology Animal Use Ethics Committee.

## Supporting Information

Figure S1 Distribution comparison of Gene Ontology (GO) categories (level 2) of transcripts among three ranid frogs, *O. margaretae*, *R. chensinensis*, and *R. kukunoris*. (TIF)

Table S1 Distribution of *O. margaretae* transcripts among KEGG pathways. (XLSX)

Table S2 Putative AMPs in *O. margaretae* sequences. (XLSX)

Table S3 SNPs in *O. margaretae* sequences. (XLSX)

Table S4 Microsatellite DNA loci in *O. margaretae* sequences. (XLSX)

Sequence S1 The final assembly of *O. margaretae* transcriptome (part 1). (RAR)

Sequence S2 The final assembly of *O. margaretae* transcriptome (part 2). (RAR)
