# Supplementary figures and images for: Transcriptome Profile of the Green Odorous Frog (Odorrana margaretae)
Source: PLoS One. 2013 Sep 20;8(9):e75211. doi: 10.1371/journal.pone.0075211 (PMC3779193; doi:10.1371/journal.pone.0075211)

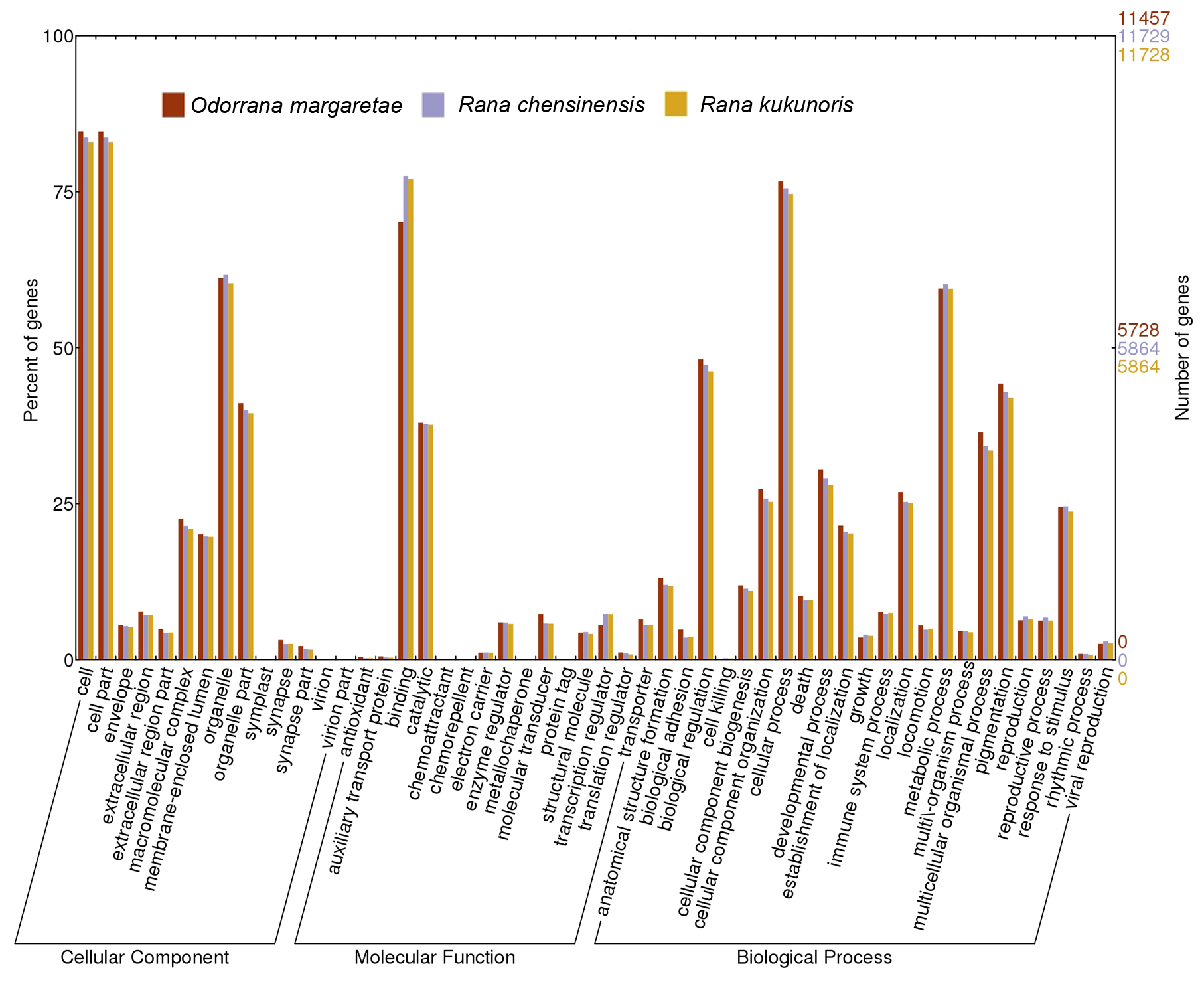

Supplement: Figure S1 — (TIF) [file pone.0075211.s001.tif]
